# Elucidating the role of a unique step-like interfacial structure of η_4_ precipitates in Al-Zn-Mg alloy

**DOI:** 10.1126/sciadv.adf7426

**Published:** 2023-06-02

**Authors:** Hwangsun Kim, Howook Choi, Juhyun Oh, Sangmin Lee, Ho Kwon, Eun Soo Park, Sungwoo Lee, Gun-Do Lee, Miyoung Kim, Heung Nam Han

**Affiliations:** Department of Materials Science and Engineering, Seoul National University, Seoul 08826, Republic of Korea.

## Abstract

Al-Zn-Mg alloys are widely used in the transportation industry owing to their high strength-to-weight ratio. In these alloys, the main strengthening mechanism is precipitation hardening that occurs because of the formation of nano-sized precipitates. Herein, an interfacial structure of η_4_ precipitates, one of the main precipitates in these alloys, is revealed using aberration-corrected scanning transmission electron microscopy and first-principles calculations. These precipitates exhibit a pseudo-periodic steps and bridges. The results of this study demonstrate that the peculiar interface structure of η_4_/Al relieves the strain energy of η_4_ precipitates thus stabilizing them. The atomistic role of this interfacial structure in the nucleation and growth of the precipitates is elucidated. This study paves the way for tailoring the mechanical properties of alloys by controlling their precipitation kinetics.

## Teaser

The formation of a unique step-like interface leads to strain relaxation of the Al matrix and η_4_ precipitates, simultaneously.

## INTRODUCTION

Recently, as carbon neutrality has become a global issue, research on high-strength lightweight alloys to increase the energy efficiency of transportation systems has attracted considerable attention (*1*, *2*). Among the existing lightweight alloys, the age-hardenable Al-Zn-Mg alloys, which are known to be aluminum alloy 7xxx series (AA 7xxx series), have been widely used in the automotive industry because of their low cost and high strength-to-weight ratio. The formation of numerous nanosized precipitates such as Guinier-Preston (GP) zones and η′ precipitates is a major hardening mechanism (*3*, *4*). This type of precipitation hardening is influenced by the type, morphology, size, and fraction of the precipitates. In particular, the morphological aspects of the precipitates are mainly attributed to their interfacial structure, which governs the mechanical properties of the alloy (*5*). As recent studies have shown that strength and ductility can be simultaneously increased because of the disordered interface structures, it is revealed that interface structure can affect not only the size and fraction of precipitates but also physical and mechanical properties (*6*). Therefore, studying the interfacial structure of the precipitates of Al-Zn-Mg alloys is fundamental for understanding the physical and mechanical properties of AA 7000 series.

The representative precipitates of Al-Zn-Mg alloys are the η-phase precipitates. The precipitation sequence of the η phase has been reported to be supersaturated solid solution (s.s.s.s.) → GP_η′_ → η′ → η (MgZn_2_) or s.s.s.s. → GP_ηp_ → η_p_ → η (MgZn_2_), depending on the alloy’s composition (*7*–*14*). Two types of GP zones have been reported for alloys (*12*). The first type is the GP I zone, which forms in solute-rich clusters from room temperature to 150°C and is fully coherent with the Al matrix. Unlike the GP zone formed in Al-Cu alloy, which was first reported separately by Guinier (*15*) and Preston (*16*) in 1938, transmission electron microscopy (TEM) and atomic probe tomography observations revealed that the GP I zone exhibits a spherical morphology (*10*, *12*, *17*, *18*). By contrast, the GP II zones, which have two types: GP_η′_ and GP_ηp_ (*13*, *19*), forms in vacancy-rich clusters of the (111)_Al_ habit plane under high vacancy concentrations; this zone can be developed when quenched above 450°C and aged above 70°C. GP_ηp_ has characteristic thickness of 7 atomic layers and double-atomic panel that is structurally stable (*13*, *19*). To date, there has been little agreement on the transformation mechanism of the GP zones to η′, and it is unclear whether η′ is generated from GP I or GP II. A recent in situ TEM study demonstrated that the GP I/Al interface can serve as a nucleation site for GP II (*20*).

η′ has a plate-like morphology with a hexagonal structure with space group *P*63/*mmc* and a nominal composition of Mg_2_Zn_5−*x*_Al_2+*x*_ (*21*–*25*). η_p_ precipitates are recently identified hardening phase with *a* = *b* = 0.496 nm and *c* = 0.935 nm (*14*). η′ and η_p_ transform to η, whose unit cell structure has a polytype (C14) of Laves phases, *a* = *b* = 0.522 nm, *c* = 0.857 nm, and space group *P*63/*mmc* (194) (*26*). There are at least 15 types of η precipitates according to their different orientation relationships with the Al matrix, and these are indicated in subscripts with numbers (*27*–*30*). Each precipitate has a different morphology and size as well as a unique interfacial structure with the Al matrix as has been revealed by TEM (*25*, *29*–*31*). Among the differently oriented η precipitates, η_1_, η_2,_ and η_4_ are the most commonly observed types in overaged Al-Zn-Mg alloys; notably, only <1% of such alloys do not have these types (*25*). Allen and Vander Sande (*31*) suggested that the η_4_ phase is the most common crystallographic variant of the η phase that forms heterogeneously on dislocations.

The formation of η improves corrosion resistance, but it also reduces the strength of the alloy with overaging (*4*). Overaging by the formation of η can have substantial effects on physical and mechanical properties, therefore controlling the microstructure and precipitation kinetics is necessary to effectively achieve desired properties, especially in AA7xxx alloy. To understand the underlying precipitation kinetics and η′ to η transformation mechanism, studies of the orientation relationships and interface structures are essential. Recently, the interfacial structure of η_1_ has been intensively studied using aberration-corrected TEM and first-principles calculations (*32*, *33*). Cheng *et al.* (*33*) presented the cosegregation of Mg and Zn atoms at the planar η_1_/Al matrix interface. Ou *et al.* (*32*) reported the periodic distribution of Zn-substitutional structural units on the η_1_/Al matrix interface viewed along [0001]_**η**1_//[1$\bar{1}$0]_Al_. However, the nucleation process of η_1_ is debated. It may nucleate from preexisting GP zones (*34*) or precipitate directly from the solid solution without any transition phases (*25*, *35*). Recent work by Zhang *et al.* (*36*) demonstrated a pathway for the formation of η_1_ via the metastable phase η_1_′. The formation of η_2_ has attracted extensive attention because of its orientation relationship with the Al matrix, which is identical to the η′ phase (*30*, *37*, *38*). The in situ high-resolution TEM observations of transformation of η′ to η_2_ proved this hypothesis (*8*).

Compared to the η_1_ and η_2_ phases, the η_4_ phase is mainly formed via heterogeneous nucleation (*31*). As the environment of the industrial sites is different from that of the typically well-controlled laboratory, heterogeneous nucleation can occur more easily. Therefore, research on the η_4_ phase is highly important for industrial applications. In addition, the recycling of Al alloys is important in terms of environmental friendliness, and the number of heterogeneous nucleation sites increases during recycling; therefore, it is important to study precipitates from the perspective of heterogeneous nucleation (*39*). However, the detailed interface structure and formation mechanism of η_4_ have rarely been studied and are yet to be elucidated. Consequently, to completely understand η_4_ formation and its impact on the overall physical and mechanical properties of alloys, an in-depth understanding of the atomic configuration of the interface is an essential prerequisite.

In this study, a unique interfacial structure of η_4_ precipitates, which are the representative precipitates for heterogeneous nucleation, was revealed, and the origin of this interfacial structure formation was elucidated using aberration-corrected scanning TEM (STEM) and first-principles calculations. The interface of the η_4_ precipitates was observed at the atomic level, which revealed that Mg and Zn gathered in small units with a bridge shape and simultaneously had a step-like overall structure. Further, the atomic configuration and origin of the formation of this unique interfacial structure were investigated.

## RESULTS

**Fig. 1.**
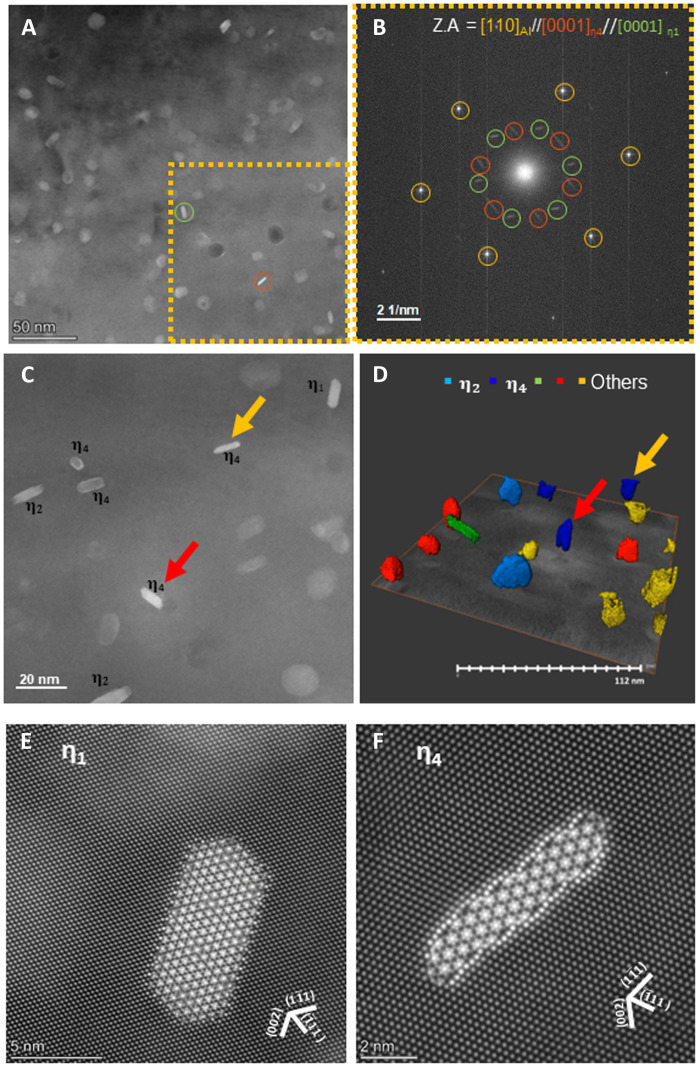
Structure and morphology of precipitates in Al-5Zn-1.5 Mg alloy. (**A**) The overall precipitate distribution of Al-5Zn-1.5 Mg alloy. (**B**) The FFT of yellow boxed area is in (A). Yellow circled peak sets are from Al, green circled peak sets are from η_1_, and red circled peak sets are from η_4_. (**C**) The STEM image of corresponding 3D tomography area of (**D**). Red and yellow arrows indicate corresponding η_4_. (**E**) Atomic-scale HAADF-STEM image of η_1_. (**F**) Atomic-scale HAADF-STEM image of η_4._

We conducted structural investigation on the precipitates in the Al-Zn-Mg alloy using TEM. The overall distribution of precipitates in the Al-Zn-Mg alloy is shown in Fig. 1A. The TEM image shows several different η precipitates with different orientation relationships. Among the observed η precipitates, those marked with red and green circles had facets, indicating the presence of a preferred orientation relationship with the Al matrix. To identify their structure and orientation relationship, we performed the fast Fourier transformation (FFT) of the yellow boxed area in Fig. 1B. The diffraction peaks from Al matrix, marked with yellow circles, indicate the [110]_Al_ zone axis. The orientation relationships between η_1_ and the Al matrix are known as (0001)_η_//(110)_Al_, $\left(10\bar{1}0\right)$_η_//(001)_Al_, whereas those of η_4_ are known as $\left(11\bar{2}0\right)$_η_//$\left(1\bar{1}\bar{1}\right)$_Al_ and (0001)_η_ //(110)_Al_ (*25*, *29*, *30*, *40*). Thus, the peaks marked with red and green circles indicate η_1_ and η_4_ precipitates, respectively. To obtain the detailed morphology of the η precipitates, we proceeded further to three-dimensional (3D) tomography. Figure 1 (C and D) shows the magnified TEM image and corresponding 3D tomography. Magnified TEM images of the [110]_Al_ zone axis and precipitates with known orientation relationship are shown in Fig. 1C. Comparing the 3D tomography result with the corresponding atomic-resolution STEM image, η_2_ and η_4_, indicated by cyan and blue colors, respectively, could be identified on the basis of the known orientation relationship. Unknown precipitates are displayed in green, red, and yellow, depending on their morphology. Red and yellow arrows indicate η_4_. Arrows with the same color indicate the same precipitate. The morphology of η_4_ was like an oval plate on {111}_Al_, which means that when the electron beam passes through the specimen along the [110]_Al_ direction, the η_4_ precipitate itself can be observed with little effect from the Al matrix. The length of the major axis of the precipitate with the red arrow was approximately 21.6 nm, which is comparable to the TEM specimen thickness.

On the [110]_Al_ zone axis, both η_1_ and η_4_ have a [0001]_η_ orientation making them appear similar. To clearly demonstrate the difference between η_1_ and η_4_, we obtained atomic-scale images of η_1_ and η_4_ along the zone axis of [110]_Al_ (Fig. 1, E and F, respectively). Because of their orientation relationship, (0001)_η_//(110)_Al_, both precipitates have the [0001]_η_ orientation, which is rotated by 24.7° along the [110]_Al_ zone axis (Fig. 1, B, E, and F). Note that the atomic configurations at the interfaces were clearly distinct. The interfacial structure of η_1_ is reported to exhibit cosegregation of Mg and Zn atoms near the η_1_/Al interface (*32*, *33*). Consistent with the literature, the observed η_1_ precipitates show segregation as indicated by the higher intensity near the interface and periodic structural subunits at the interface. However, the interfacial structure of η_4_/Al has rarely been studied; therefore, we focused on this interface.

**Fig. 2.**
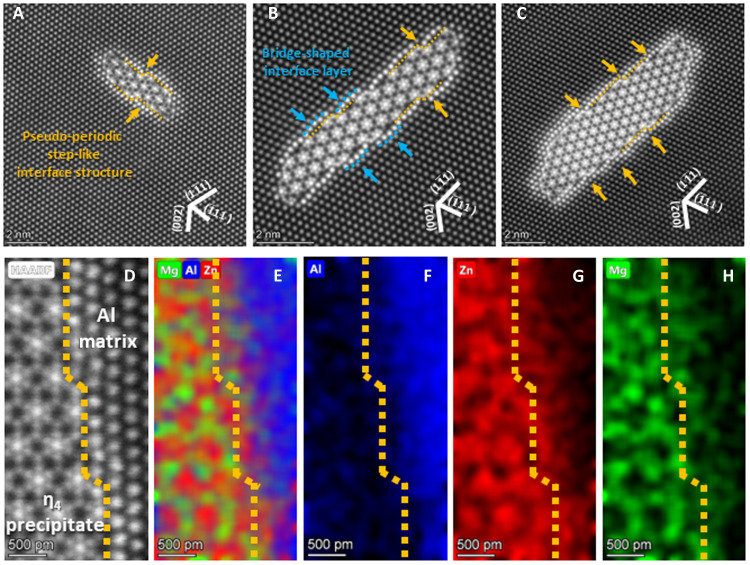
Atomic scale observation of η_4_ precipitates. (**A** to **C**) Atomic-scale HAADF-STEM image of η_4_ having different sizes. Blue dotted line in (B) shows bridge-shaped interface of the η_4_/Al. (**D** to **H**) HAADF-STEM and corresponding EDS mapping results obtained at the interface of η_4_ /Al. (D) HAADF-STEM image of reference area. (E) Al, Mg, and Zn maps overlapped together. (F) Al map (G) Zn map, and (H) Mg map. Yellow dotted line indicates the pseudo-periodic step-like interface of η_4_ and Al matrix.

We further investigated the atomic configurations of η_4_ precipitates using high-resolution STEM imaging. Previous studies on η_4_ mostly focused on the analysis of the diffraction patterns or conventional bright-field/dark-field TEM images (*25*, *31*). High-resolution high-angle annular dark-field STEM (HAADF-STEM) images provided additional information, particularly near the η_4_/Al interface. Figure 2 (A to C) shows η_4_ precipitates of different sizes. Regardless of the precipitate size, the atomic column intensity near the η_4_/Al interface is higher than that of the Al matrix. This suggests a higher Zn concentration near the interface given the Z-dependent contrast of the HAADF images. The yellow dotted lines in Fig. 2 (A to C) indicate the boundary between η_4_ and Al, and the blue dotted line traces the bridge-shaped interfacial layer, revealing its slightly curved nature rather than straight lines. Notably, in these HAADF-STEM images, is that in contrast to η_1,_ which has a planar interface, η_4_/Al has an interface with pseudo-periodic steps and bridges. In general, the step or ledge-shaped interface of the precipitates is accompanied by their growth (*41*, *42*). However, in contrast to the commonly observed precipitates, the steps marked with the yellow dotted line shown in Fig. 2 (A to C) are nearly periodic regardless of their size, indicating that they are inherent to the interfacial structure rather than associated with precipitate growth. To identify the chemical composition of this unique interfacial structure more precisely, we performed with atomic-scale energy-dispersive x-ray spectroscopy (EDS) and obtained the elemental composition of each atomic column (Fig. 2, D to H). Al, Zn, and Mg atoms are indicated by blue, red, and green, respectively. As shown in Fig. 2F, Al atoms barely existed near the interface. By contrast, Mg and Zn were concentrated near the η_4_/Al interface, including the bridge-shaped interfacial layer (Fig. 2, G and H). These results suggest that η_4_ precipitates have a unique pseudo-periodic step-like interfacial structure consisting of Mg and Zn segregation layer.

In addition, we examined the HAADF-STEM images, whose intensity is proportional to Z^1.7^ (*43*–*48*), for the quantitative analysis of the interfacial structure because it is hard to get additional atomic-scale information from other zone axis (fig. S1). CalAtom software was used to determine the position and average intensity of each atom column: the Al matrix, η_4_ precipitates (composed of Mg and Zn), and η_4_/Al interface (*49*). Initial structural models were constructed on the basis of these experimental results, which were further explored to determine a stable interfacial structure using first-principles calculations.

**Fig. 3.**
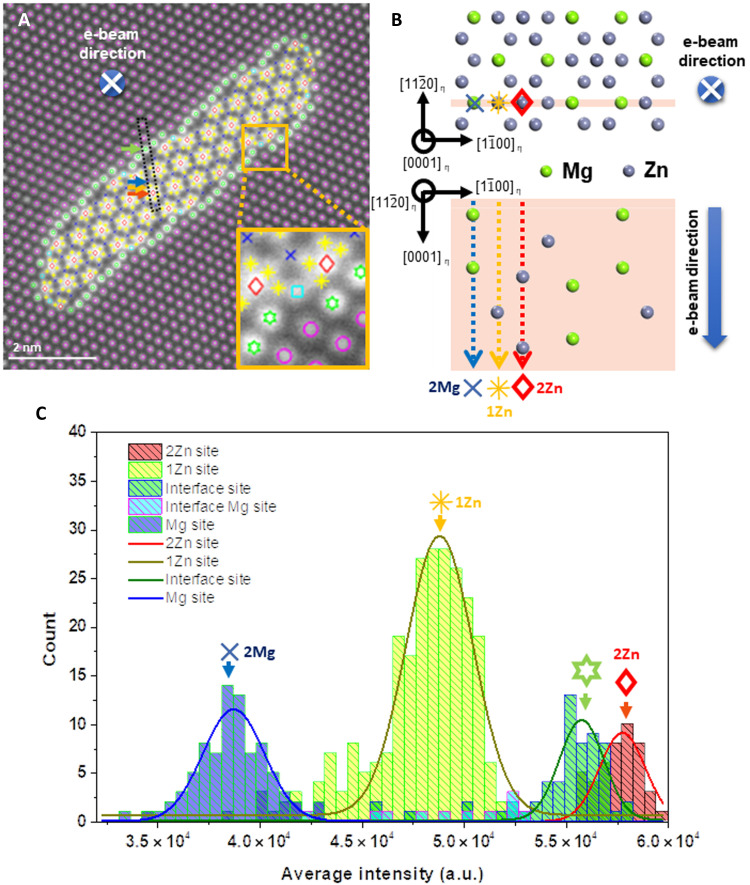
Atomic column intensity distribution of η_4_ and Al. (**A**) Similar atomic column densities are grouped and marked with different symbols. Red rhombus indicates 2Zn site, yellow star indicates 1 Zn site, blue x indicates 2 Mg site, green hexagon star indicates the interface layer (bridge) site, cyan square indicates the interface layer (step) site, and magenta circles indicate Al matrix. (**B**) Schematic diagram of atomic model of η precipitates. (**C**) Average atomic column intensity profile of (A). a.u., arbitrary units.

For quantitative analysis, atomic columns with similar intensities were grouped and displayed with different colors and shapes, as shown in Fig. 3A. The yellow boxed area shows six groups with distinctive intensities: the Al matrix (magenta circle), the bridge-shaped interface layer (green hexagon),the step-shaped interface layer (cyan square), and three different groups in the η_4_ precipitate (red rhombus, yellow star, and blue x-shaped mark). Figure 3B shows a schematic of the η_4_ precipitate with different crystallographic orientations. The same marks in Fig. 3A are indicated at the corresponding position. The upper image of Fig. 3B exhibits a 2D-projected view along the [0001]_η_ direction, which is the same as the incident electron beam direction shown in Fig. 2. The lower image is viewed along the 90° rotated [11$\bar{2}$0]_η_ zone axis. The orange-background planes in the upper and lower schematics in Fig. 3B represent the same area. In the (0001)_η_ orientation, it may be misleading that the intensities of all Zn atomic columns are the same from the schematic. However, the line densities of Zn and Mg atoms are different as shown in the schematic of the [11$\bar{2}$0]_η_ zone axis; the relative atomic number ratio of each marked group is 2 Zn (red rhombus): 1 Zn (yellow star): 2 Mg (blue x-shaped mark). The HAADF-STEM image (Fig. 3A) demonstrates a substantial intensity difference between the red diamond and yellow star sites owing to the different elemental densities along the [0001]_η_ direction.

The intensities of the atomic columns in the HAADF-STEM images could provide insight for demonstrating the interfacial structural model. First, we focused on the bridge-shaped interfacial layer (marked with a green hexagon): The average intensity distribution of each atom column is shown in Fig. 3C. The intensity distribution of each group was fitted using the Gaussian curve fitting method. This intensity profile shows that the atomic column intensity of each column in the interfacial layer, particularly in the bridge-shaped layer (indicated by the green hexagon), is uniform. In addition, the average intensity of the interfacial layer was found to be between those of the yellow star (1 Zn) and red rhombus (2 Zn). Combining this with the EDS results, the interfacial atomic layer is composed of the combination of Mg and Zn, and the atom column density of the bridge-shaped interfacial layer was between those of 1 Zn and 2 Zn. In addition, Fig. 2 (A to C) shows that the structure of the interfacial layer was similar to that of the Al matrix. On the basis of the EDS and HAADF-STEM image analysis results, various models were created by substituting one Zn atom and two Mg atoms for each atomic column at the interface, and the total energies were compared after relaxation through first-principles calculations (figs. S2 to S5). From the calculated results, two different models were established: a planar interfacial model with a bridge-shaped interfacial layer and a step-like interfacial model consisting of a bridge-shaped interfacial layer and a stepped interfacial structure.

**Fig. 4.**
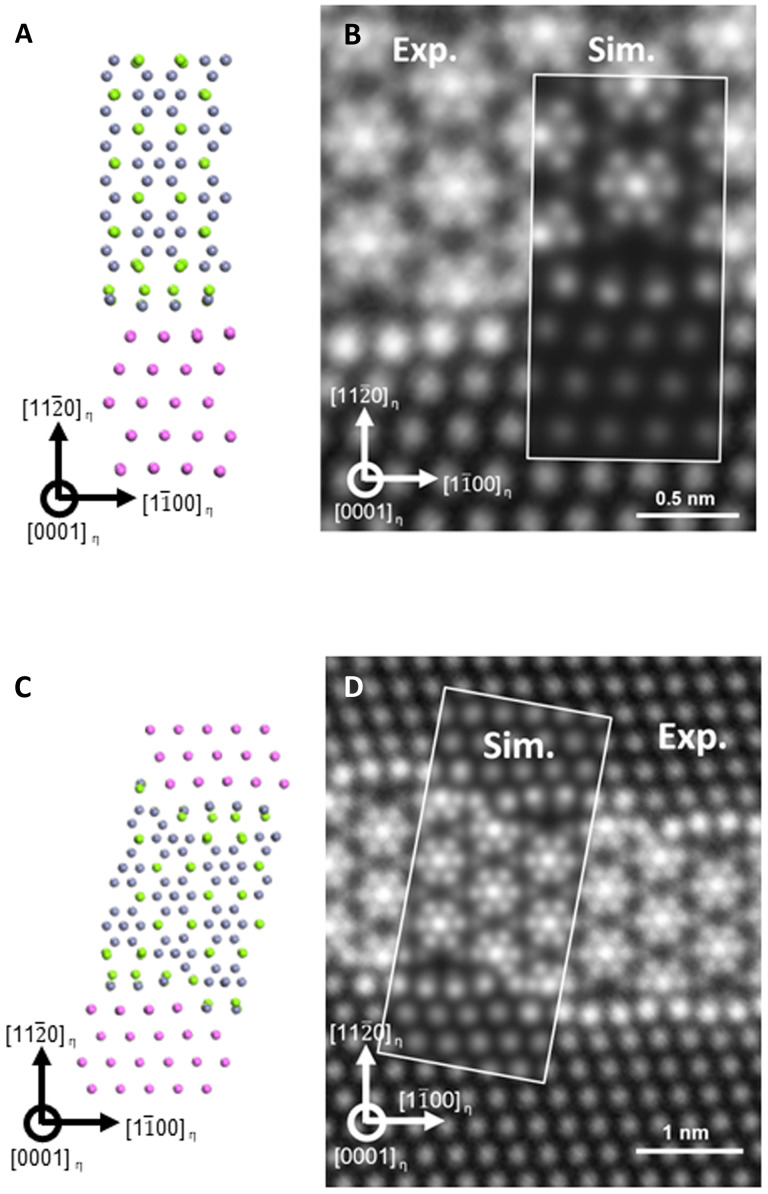
First-principles calculation result and corresponding STEM simulation of planar interface model and step-like interface model. (**A**) Schematic diagram of atomic model of η/Al having planar interface. (**B**) HAADF-STEM image and corresponding simulation of (A). (**C**) Schematic diagram of atomic model of η/Al having step-like interface. (**D**) HAADF-STEM image and corresponding simulation of (C).

To elucidate the origin of the step-like interface formation, we compared two different models: the planar interfacial model and the step-like interfacial model that was constructed on the basis of on the observed images. Schematics of the most stable structures of the planar and step-like interfaces among the calculated models are displayed in Fig. 4 (A and C, respectively). The periodicity of each model is shown in fig. S6. The calculated models describe the bridge-shaped interfacial layer well, mainly with the arrangement of Mg and Zn atoms in a slightly curved form. STEM image simulations were performed using the calculated models, and the results are shown in Fig. 4 (B and D) together with the observation results. The simulations on Fig. 4B has slight difference from the observation results along $\left[1\bar{1}00\right]$_η_//[1–10]_Al_. However, the STEM image simulation (Fig. 4D) of this optimized structure successfully reproduced the bridge-shaped interface of the experimentally observed STEM images within 2% of error, suggesting that the interfacial structure was accurately determined considering the larger pixel size of the simulated image. The effect of vacancy was also considered with possible models, but none of them were energetically favored (fig. S7).

**Fig. 5.**
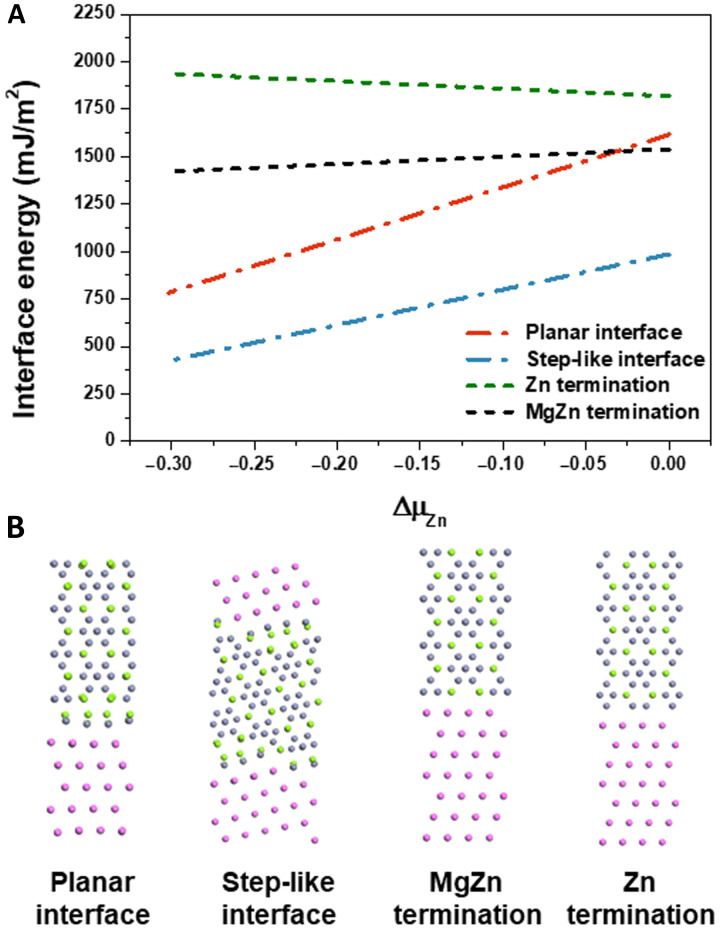
Interface energy of possible interfacial structure. (**A**) Calculated η_4_ /Al interface formation energy of each model. (**B**) Schematic diagram of each model: planar interface, step-like interface, MgZn termination, and Zn termination.

To confirm the stability of this unique interfacial structure, we compared the interfacial formation energies with four different terminations: a planar interface without the stepped structure, a step-like structure, and two artificial interfacial structures without the interfacial layer (Fig. 5). Two different artificial models were labeled as MgZn termination and Zn termination, considering the termination of the η_4_ precipitates. In the MgZn termination model, Mg and Zn atoms meet the Al matrix, whereas in Zn termination, only Zn atoms meet the Al matrix. The total energy of each model was calculated using the first-principles calculations. The formula used for the calculations is as follows (*32*, *50*)

$$\gamma =\frac{1}{2A}(E_{\mathrm{tot}}-N_{\mathrm{Al}}\mu_{\mathrm{Al}}-N_{\mathrm{Mg}}\mu_{\mathrm{Mg}}-N_{\mathrm{Zn}}\mu_{\mathrm{Zn}}+P\text{Δ}V-T\text{Δ}S)$$ (1)

where γ is the interfacial energy and *A* is the cross-sectional area of the interface. The cross-sectional area of the step-like interface was calculated by adding the areas of all adjacent interfaces (yellow dotted line in Fig. 2D). *E*_tot_ is the total energy of the fully relaxed interface supercell. *N_i_* and μ*_i_* are the number and chemical potential of element *i* (*i* = Al, Mg, or Zn), respectively. ∆*V* is the volume change due to interfacial relaxation, and ∆*S* is the vibrational entropy difference due to interface formation. *P*∆*V* and *T*∆*S* are relatively small and can be disregarded under ambient pressure and a practical aging temperature of 100° to 200°C, which is far below the melting point (~660°C).

To calculate the interfacial formation energy of Al/η_4_, the bulk formation energy of the η_4_ precipitate should be considered (*32*, *50*). When the Al/η_4_ interface reaches its thermal equilibrium, ∆*E* = 0 for η formation or decomposition, then the sum of elemental chemical potentials is equal to the chemical potential of η. For a pure substance, μ_η_ = μ°_η_, where superscript o stands for the standard state. Thus

$$\mu_{\mathrm{Mg}}+2\mu_{\mathrm{Zn}}=\mu_{\eta }=\mu_{\eta }^{o}$$ (2)

The chemical potential of η can also be expressed using the chemical potential of the standard state.

$$\mu_{\mathrm{Mg}}^{o}+2\mu_{\mathrm{Zn}}^{o}+\text{Δ}H_{\eta }^{o}(\eta )=\mu_{\eta }^{o}$$ (3)

∆μ*_i_* defines the excess chemical potential of element *i* and represents the deviation in the real chemical potential μ*_i_* at the interface from its standard state value, that is, ∆μ*_i_* = μ*_i_* − μ°*_i_*. Because of the large cancellation among the temperature dependences of all energy terms *E*_tot_ and μ*_i_* in Eq. 1, as a first-order approximation, all the total energy terms *E*_tot_ and μ°*_i_* can be estimated by 0-K enthalpy calculations. Thus, the temperature dependence of the interfacial energy predominantly relies on ∆μ_Zn_(= *kT* ln *a*_Zn_). The interfacial formation energy can be rewritten by combining Eq. 1 with Eqs. *2* and *3*

$$=\frac{1}{2A}[E_{\mathrm{tot}}-N_{\mathrm{Al}}\mu {}_{\mathrm{Al}}^{\circ }-N_{\mathrm{Mg}}\mu {}_{\eta }^{\circ }-(N_{\mathrm{Zn}}-2N_{\mathrm{Mg}})\mu {}_{\mathrm{Zn}}^{\circ }-(N_{\mathrm{Zn}}-2N_{\mathrm{Mg}})\text{Δ}\mu_{\mathrm{Zn}}]$$ (4)

To ensure a stable interface, the chemical potential of Zn and Mg at the interface must be limited to less than that in the pure bulk standard state to ensure a stable interface. Thus, ∆μ_Zn_ and ∆μ_Mg_ were less than zero. In addition, the formation energy of the bulk η phase in Al is

$$\text{Δ}H_{f}^{{}^{\circ }}(\eta )=\text{Δ}\mu_{\mathrm{Mg}}+2\text{Δ}\mu_{\mathrm{Zn}}$$ (5)

Thus, the reasonable varying range of ∆μ_Zn_ can be determined as

$$\frac{1}{2}\text{Δ}H_{f}^{{}^{\circ }}(\eta )<\text{Δ}\mu_{\mathrm{Zn}}<0$$ (6)

The formation energy of bulk η is calculated to be −0.60 eV per formula unit. Using Eqs. 4 and 6, all interfacial energies can be calculated with respect to ∆μ_Zn_, which is in between −0.30 and 0 eV. Figure 5A shows the interfacial formation energy of each model depending on the changes in ∆μ_Zn_. Notably, although the formation of a step-like interface is not a common phenomenon, it is energetically preferred, regardless of the changes in ∆μ_Zn_.

The interfacial energy is affected by two factors: chemical contributions due to compositional changes and structural contributions due to the structural distortions caused by misfit strain (*51*). Regardless of the interface type, the chemical composition distributions were almost identical. In the case of structural contributions, neither planar interface nor step-like interface do not consist of dislocations or other structural distortions. Therefore, we focused on the structural contributions of misfit strain.

**Fig. 6.**
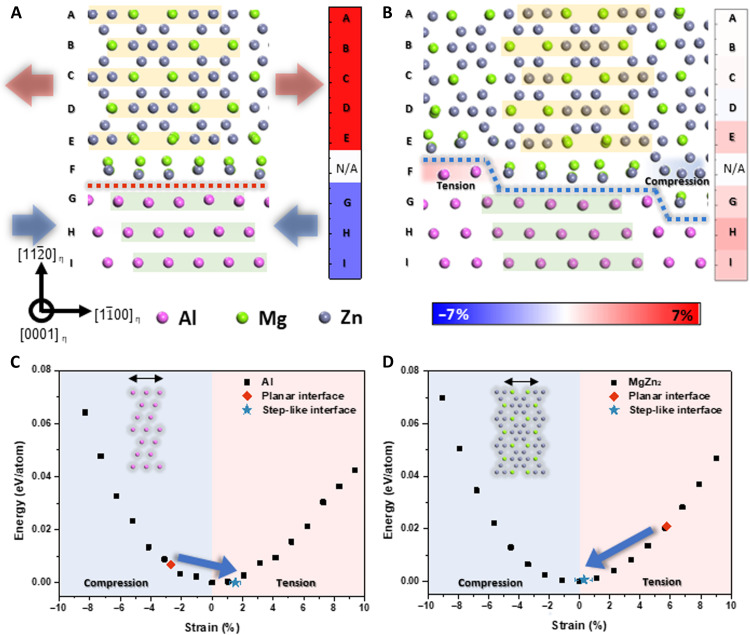
Strain energy of planar and step-like interface. Schematic diagram of simulated interfaces. (**A**) Planar interface and (**B**) step-like interface. (**C**) Strain energy of Al matrix along [1$\bar{1}$0]. (**D**) Strain energy of η precipitates along [1$\bar{1}$00]. Error bars of step-like interface in (C) and (D) show the SD of the measured strain from the observation.

On the basis of the calculated model in Fig. 4, we measured the interatomic distances of the η precipitate and Al matrix to confirm the structural contributions caused by the misfit strain. Figure 6 (A and B) shows the schematics of η/Al with a planar interface and η/Al with a step-like interface. We measured the interatomic distance of each layer assigned with capital letters. The A to E layers indicate the atomic layers of the η precipitate, the F layer indicates the interface, and the G to I layers indicate the Al matrix. The color bar on the right side shows the strain level of each layer: red for tensile strain and blue for compressive strain. The η precipitate in the planar interfacial model in Fig. 6A underwent tensile strain, whereas that of the Al matrix underwent compressive strain. By contrast, the strain of the η precipitate and Al matrix in the step-like interface was relaxed compared to that in the planar interface. In addition, the geometrical phase analysis (GPA) results in fig. S9 also show that the strain of the Al matrix near the η precipitate is about ±2%.

We additionally calculated the energy difference of η precipitate and Al matrix with increasing tensile and compressive strain along $\left[1\bar{1}00\right]$_η_ direction. Figure 6C shows the energy difference of Al matrix, and Fig. 6D indicates the energy difference of the η precipitate. The energy of the corresponding measured strain is marked in Figs. 6 (C and D) with red diamonds (planar interface) and blue stars (step-like interface). The strain energy of the Al matrix at the step-like interface was slightly lower than that at the planar interface. The strain energy of the η precipitate of the step-like interface was also drastically reduced compared to that of the planar interface. Therefore, we can conclude that the formation of the step-like interface induces strain relaxation of the η precipitates and Al matrix, which is energetically favored considering its strain energy.

## DISCUSSION

When two arbitrary materials form an interface, how the detailed structure of the interface is formed can be mathematically explained using the O-lattice theory (*52*). Coincidence site lattice boundary could be also predicted using this theory (*53*). However, because this theory assumes that two different materials themselves are perfect crystals, it has the disadvantage that atomic relaxation near the interface cannot be considered. In our study, we tried to identify the origin of the stepped interface formation based on actual observation results using an atomic-resolution TEM.

When the misfit strain is small, the matrix and precipitates maintain coherency. However, as it increased, the matrix and precipitate began to lose coherency. This may have occurred after the growth of the precipitates. The misfit strain of η_4_/Al was approximately 9% comparing interatomic distances. Considering this misfit strain value, if η_4_/Al maintained a planar interface, then it was expected to cause many dislocations after the growth of η_4_. However, as shown in Fig. 2C, despite the fairly large misfit strain value of 9%, coherency was maintained after precipitate growth was completed. Moreover, the GPA result in fig. S9 also indicates that the strain value near the eta precipitates is under 2%. This may explain the role of the stepped structure in reducing the strain energy.

What then causes the step-like interface to reduce the misfit strain? The structural difference between the planar and step-like interfaces clearly demonstrates the reason for the strain relaxation. As shown in Fig. 6A, the η precipitate and Al matrix are subjected to tensile and compressive strains, respectively, to fit each other along [1$\bar{1}$00]_η_. The Al matrix then tends to expand and the η precipitate prefers to contract to relieve strain. In this case, Fig. 6A shows that each atomic layer, denoted by a capital letter, exists independently without mixing with other substances. By contrast, Fig. 6B shows that in the step-like interfacial structure model, there is an F layer in which Al, η, and a bridge-shaped interfacial structure coexist along the $\left[1\bar{1}00\right]$_η_ direction. The interatomic distance of η in the F layer was the same as that in the E layer, and that of Al in the F layer was identical to that of the G layer considering their periodicity. As shown in the color bar on the right side of Fig. 6 (A and B), the extended η precipitate structure near the bridge-shaped interfacial layer was relaxed with compression, and the condensed Al near the bridge-shaped interface layer was relieved with tension compared with the planar interface. As mentioned previously, Al can relieve strain with extension, and η precipitates can relieve strain with contraction. The bridge-shaped interface layer of the step-like interface could serve as a buffer zone, relaxing both the Al matrix and η precipitates compared to those in the planar interface.

Notably, compared with other generally observed precipitates with planar interfaces, the precipitation of η_4_ precipitates was accompanied by an increase in the interface area because of the step-like interface. To become more stable despite the increasing area of the interfacial layer, the interface formation energy must be much smaller than the increase in the interface area. The interface formation energy of the planar interface was approximately two times larger than that of the step-like interface (Fig. 5A). The interface area of the step-like interface was ~27% higher than that of the planar interface. Overall, the total interface formation energy decreased by ~37% with the formation of the step-like interface.

On the basis of the results and discussions above, we can lastly elucidate the reason for the formation of the step-like interface. Although the step-like interface accompanies the expansion of the cross-sectional interface area, the effect of the strain energy reduction compensates for the additional increase in the interface energy. From the strain energy graph (Fig. 6, C and D), we can observe that in the planar interface, Al is constrained and η_4_ precipitates are expanded compared to their most stable states. In contrast to the planar interface, the step-like interface had a buffer layer that coexisted with the Al matrix, bridge-shaped interfacial layer, and η precipitate. The presence of the bridge-shaped interfacial layer creates a margin space; consequently, Al expanded and η_4_ precipitates shrunk such that they could be rearranged in a direction where they could be stable.

In summary, the unique step-like interfacial structure of η_4_/Al in Al-Zn-Mg alloys and its structural origin were studied by atomic-resolution HAADF-STEM, EDS, STEM simulation, and first-principles calculations. Using 3D tomography, η_4_ precipitates with oval plate morphology lying on {111}_Al_ were observed. The atomic configurations near the η_4_/Al interface were revealed to be the cosegregation of Mg and Zn. Pseudo-periodic bridges and steps were detected at the η_4_/Al interface, regardless of the size of the η_4_ precipitates. Their atomic structure was revealed using first-principles calculations and subsequently verified by STEM simulations. The first-principles calculations showed that the step-like interface had the lowest interface formation energy, implying that it was energetically favored. Furthermore, the formation of a stepped structure at the interface mediated the simultaneous decrease in the strain energies of Al and η_4_. Therefore, both the Al matrix and η_4_ precipitates could coexist in a relatively stable state, forming a step-like interface. Our elucidation of the mode for creating stable interface with a unique interfacial structure could pave the way for tailoring the mechanical properties of alloys by controlling the precipitation kinetics.

## MATERIALS AND METHODS

### Specimen preparation

The Al-5Zn-1.5 Mg alloy was fabricated by vacuum induction melting to eliminate the effect of other alloying elements in the formation of η precipitates. Small samples cut from the ingots were homogenized at 460°C for 24 hours, followed by water quenching to room temperature and cold rolling to sheets of 0.5-mm thickness. The sheets were solution-treated at 460°C for 1 hour and water-quenched, and η precipitates were formed after pre-aging at 100°C for approximately 5 hours and aging at 150°C for approximately 6 hours. The quench procedure might cause large deformations in the thin specimens. Therefore, a kernel average misorientation map using electron back-scattered diffraction was acquired to check the deformed state after solution-treated followed by water quenching (fig. S10). The data acquisition step size for each Kernel average misorientation (KAM) analysis is 0.2 μm. Because cleaning up the electron backscatter diffraction data can change KAM value substantially, the cleanup was not performed. Instead, data pixels with an absolute value of confidence index of 0.1 or less were excluded from the analysis by unindexing. There was no notable deformation that could affect the TEM observation. Thin TEM specimens were prepared by cutting discs into 3-mm pieces and mechanically thinning the discs to 0.07 mm before using a twin jet electro-polisher at −25°C with a working voltage of 11 V. The electrolyte was composed of 33% nitric acid and 67% methanol.

### Image acquisition and analysis

HAADF-STEM images were acquired using an image and probe Cs-corrected Thermofisher Themis Z operated at 300 kV, and EDS images were obtained at 200 kV. Each atomic-resolution STEM image was collected with 100 ns per image, and 20 images were used for drift-corrected frame integration to remove the effect of possible drift and scanning beam distortions. The convergence semiangle and inner collection angle were 30 and 50 mrad, respectively. STEM simulations were performed at 300 kV using Dr.Probe software (*54*). Spherical aberration coefficients of Cs = −1.32 μm and C5 = 587 μm; a convergence semiangle of 30 mrad; and a slice thickness of 8 Å were set during the simulations. The thickness of the specimens was set to 40 nm.

### First-principles calculation

First-principles calculations were performed using the Vienna ab initio simulation package. The projector-augmented wave method with local density approximation was used. For all calculations, an energy cutoff of 400 eV was used for the plane-wave basis set expansion. The K-points are set as 4 × 4 × 1 for planar models and 6 × 4 × 1 for stepped models. The ground-state atomic structures were obtained by minimizing the Hellman-Feynman forces until the total forces on each ion converged to within 0.02 eV/Å. To calculate strain energy, the superlattice size along $\left[1\bar{1}00\right]$_η_ and [1$\bar{1}$0]_Al_ were changed within −10% to +10%, and the corresponding energies were compared with their energetically favored model, Al with Al and η with η.
