# Supplementary material for: Elucidating the role of a unique step-like interfacial structure of η4 precipitates in Al-Zn-Mg alloy
Source: Sci Adv. 2023 Jun 2;9(22):eadf7426. doi: 10.1126/sciadv.adf7426 (PMC10413671; doi:10.1126/sciadv.adf7426)
Supplement: Supplementary file 1 — Supplementary Text Figs. S1 to S10 [file sciadv.adf7426_sm.pdf]

Science Advances
AAAS

Supplementary Materials for

Elucidating the role of a unique step-like interfacial structure of η_4 precipitates in Al-Zn-Mg alloy

Hwangsun Kim *et al.*

Corresponding author: Heung Nam Han, hnhan@snu.ac.kr; Miyoung Kim, mkim@snu.ac.kr; Gun-Do Lee, gdlee@snu.ac.kr

Sci. Adv. **9**, eadf7426 (2023)
DOI: 10.1126/sciadv.adf7426

This PDF file includes:

Supplementary Text
Figs. S1 to S10

Supplementary Text

The observation along <112>$_{Al}$ zone axis

To identify the interfacial structure of η_4 precipitates, we additionally performed the observation along <112>$_{Al}$, which is 90 degrees rotated from the <110>$_{Al}$ and expected to have additional atomic-scale information of the η_4/Al interface. However, as shown in Fig. S1, it is difficult to resolve the interface structure and hard to get rid of the overlap effect of the Al matrix considering its 3D morphology, TEM specimen thickness and pseudo-periodic stepped interface structure. Nevertheless, the STEM simulation result based on the step-like interface model along <112>$_{Al}$ matches well with the observation results.

The method for the formation of first-principle calculation model for planar and step-like interface models

Planar interface model

A structural model of the η_4 and Al interface for the first-principles calculations is shown in Figs. S2(a) and (b). Two atomic layers near η_4/Al interface is marked with orange background and the interface layer which is initially Al matrix is marked with blue background. Figs. S2(c) and (d) are planar pictures of colored layers in Fig. S2(a). Assuming that the precipitates and interface layer have the same thickness, the interface layer (green hexagon) have 3 atoms when red rhombus position in Figs. S2(a) and (c) have 2 Zn atoms through electron beam direction. As was mentioned before, the atom column density of the interface layer was between 1 Zn and 2 Zn, composed of the mixture of Mg and Zn. In order to satisfy all these conditions, each atom column of the interface should be composed of 1 Zn and 2 Mg in the model shown in Figs. S2(a) and (d). Various models were created by substituting one Zn atom and two Mg atoms for each atomic column at the interface (Fig. S2 and S3), and the total energies were compared after relaxation through first-principle calculations (Fig. S4).

Step-like interface model

Step-like interface model was basically established based on the planar interface model which well described bridge-shaped interface layer. One different thing is that step-like interface model have junction (cyan square) as shown in Fig. 3A. To address this junction layer, we look into the experimental STEM observations. The shapes and colors in Fig. S5(a) are identical to those in Fig. 3. The colored arrows in the yellow box area in fig. S5(a) indicates equivalent 2 Mg lattice sites. However, the cyan colored arrow shows that the atom column intensity of the junction of stepped shape interface site (cyan square) is significantly different from other 2 Mg sites (blue x-shaped mark site) despite the equivalent position with 2 Mg lattice sites. To quantify the atomic column of the junction of stepped interface, the line intensity profile of the white dotted box in Fig. S5(a) is shown in Fig. S5(b). The atom column intensity of the junction of stepped shape interface was between 1 Zn and 2 Zn. Since the number of the junction of stepped shape interface site in Fig. 3(a) was 6, which was not enough for the quantification using Calatom software, we compared 20 different sites of the junction of stepped shape interface site with different images of η_4. Among the 20 different sites, 19 sites show the same tendency with the intensity profile shown in Fig. S5(b). Considering the observation result of stepped shape interface in Figs. 3A and S5(a), 3 possible initial model was constructed for the first-principle

calculations. (Figs. S5(c) to (e)) The base model, of which the junction of the stepped shape interface lattice site is consisted only with Mg, is constructed with Al matrix and previously obtained bridge shape interface structure with first principle calculations. Here, the atomic column marked by cyan square is assigned as 2 Mg, extended from the η phase. The red dotted circle areas in Figs. S5(d) and (e), initially 2 Mg site, were replaced with 1 Mg atom and 1 Zn atom to reflect the intensity profile in Fig. S5(b). The difference between two models, Step mod1 and Step mod2, is the position of substituted Zn atom. The total energy calculations show that Step mod1 is the most stable structure. Since the atomic number of base model is different with the Step mod1 and Step mod2, the chemical potentials of Zn metal and Mg metal are used to adjust different number of atoms in each structure.

Comparing calculated energy having different atomic configurations
The total energies of these three different models from step-like interface, are shown in Fig. S5(f). Comparing base model with Step models, total number of atoms are same but Step models have 2 Zn atoms more and 2 Mg atoms less than base models. Thus, the substitution energy should be considered. The chemical potential of Mg and Zn atoms are -1.826 and -1.891 eV/atom each. The difference between the chemical potential of Mg and Zn is about 0.065 eV/atom. Two Mg atoms are substituted with two Zn atoms in Step models. Therefore, if the Step models have more than 0.13 eV lower than the base model, they are more energetically favored compared with base model. The condition for base model to be stable is follows:

$$E_{tot,base} - 2\mu_{Mg} + 2\mu_{Zn} < E_{tot,Step}$$
$$E_{tot,base} - 0.13\ eV < E_{tot,Step}$$

The total energy of base model was -717.8 eV and the Step mod1 was -718.2 eV. The total energy difference between base model and Step mod1 is about 0.4 eV, which is higher than the substitution energy, 0.13 eV. This infers that the Step mod1 is the energetically favored compared with the base model.

Periodicity of the planar and step-like interface model
Performing first-principle calculation and image simulation requires the periodicity of the supercell structure. To build the periodic planar interface model, we measured the periodic units with the least common multiple of Al and η along $[1\bar{1}00]_{\eta}$ direction. In case of the step-like interface model, we rotated the supercell about 15 degrees compared with the planar model to make the stepped structure to be periodic. The periodic supercell structure of the planar and step-like interface model is displayed in Fig. S6.

Effect of vacancy at the bridge-shaped interface layer
In the HAADF-STEM images of η_4/Al, it is difficult to distinguish whether there are vacancies at the interface, especially near the bridge-shaped interface layer (Figs. 1F, 2A – C). To demonstrate the effect of the vacancy, we made two arbitrary models based on the step-like interface model (Fig. S7). The first is Vac1, which has 4 vacancy sites that are expected to be present in the bridge-shaped interface layer. In this case, we removed 4 Mg atoms from the step-like interface model. The second is Vac2, which has 10 vacancy sites considering the uniformity of the atomic column intensity (Fig. 3). In this case, we removed 10 Mg atoms from the step-like interface model. Each atomic column of the bridge-shaped interface layer in the Vac2 model

consists of 1Zn and 1Mg. The ground-state atomic structures of Vac1 and Vac2 models were obtained with the same first-principle calculation condition with the step-like interface model. We compared the total energy of the Vac1 and the Vac2 model with the step-like interface model. The total energy of the Vac1 model was 0.907 eV and 0.003 eV/atom higher than the step-like interface model. In case of the Vac2 model, the total energy was 9.607 eV and 0.026 eV/atom higher than the step-like interface model. Both Vac1 and Vac2 models were energetically unfavored compared with the step-like interface model. We also performed the STEM image simulations and with Vac1 and Vac2 models, and the results are shown in Fig. S7. The intensity distribution near the bridge-shaped layer is inhomogeneous, which is significantly different from the TEM observation results. Based on the first-principle calculation and STEM image simulation, it can be concluded that the presence of vacancies at the interface is energetically unfavorable, even though vacancies appear to exist at the interface.

Strain analysis of the observation result using GPA analysis

To calculate the strain from the observation results, GPA analysis was additionally performed using the HAADF-STEM image (Fig. S8). The Al lattice was used as the reference for carrying out the strain analysis. The strain map in Fig. S8, ε_{xx}, shows the strain of Al along the $[1\bar{1}00]_{\eta}$ direction, and the underlying strain profile with the blue arrow indicates that the strain near the η_4/Al interface is under 2%, which agrees well with the strain calculated from first-principle calculation.

Effect of Al on the quantitative analysis with HAADF-STEM

There is still considerable uncertainty with regard to the point that whether observed HAADF-STEM image of η_4 precipitates contain Al matrix through e-beam direction or not. The results shown in Fig. S9 are obtained under the assumption that precipitates occupy a significant amount compared to the Al matrix in the direction of the incident electron beam. Therefore, it is necessary to understand the effect of the Al on the quantitative analysis. To get the knowledge of the effect of additional Al matrix through e-beam direction, the artificial model having four different fraction of Al matrix through e-beam direction; 0 %, 25 %, 50 %, 63 % was made. These four models are labeled as Al 0 %, Al 25 %, Al 50 %, and Al 63 % each. STEM simulation was performed with 4 models having different amount of Al matrix (Fig. S9(a) to (d)). When the proportion of Al is less than 50%, the simulated image is similar to the Fig. 2B and C. When it exceeds 50%, it can be confirmed that it is similar to Fig. 2A. We also compared the intensity profile of each model in the same area in Fig. 3A (same with the Fig. S9(e)). The intensity profile of Al 0% and Al 25% has a similar tendency compared with Fig. S9(g). All these results imply that the image used to elucidate the interfacial structure of η_4 (Fig. 2B) appear to lie between 0% and 25% Al. The fact that the η_4 precipitates have oval plate shape, of which the size of the main axis is comparable with TEM specimen thickness (Fig. 1D) also supports this result. Besides, EDS results in Fig. 2F also shows that Al atoms are barely exist near and inside of the η_4 precipitates, meaning that η_4 precipitates exist itself without containing the Al matrix along electron beam direction. Therefore, the influence due to the presence of Al matrix is negligible.

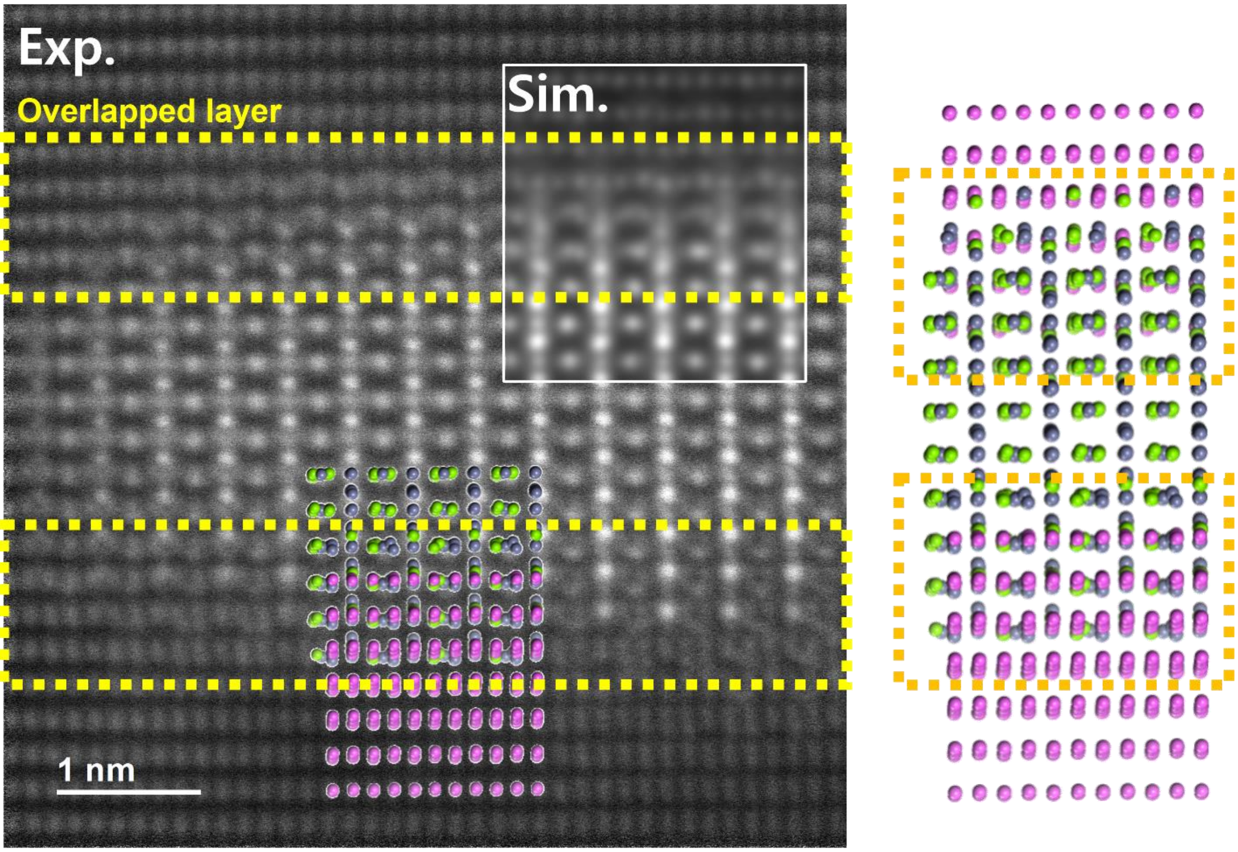

Fig. S1. The atomic-scale HAADF-STEM observation along <112>$_{Al}$. The yellow dotted boxes indicate the overlapped layer of Al matrix, η_4 precipitate, and η_4/Al interface structure. The STEM simulation result from the step-like interface structure model was indicated with the white box.

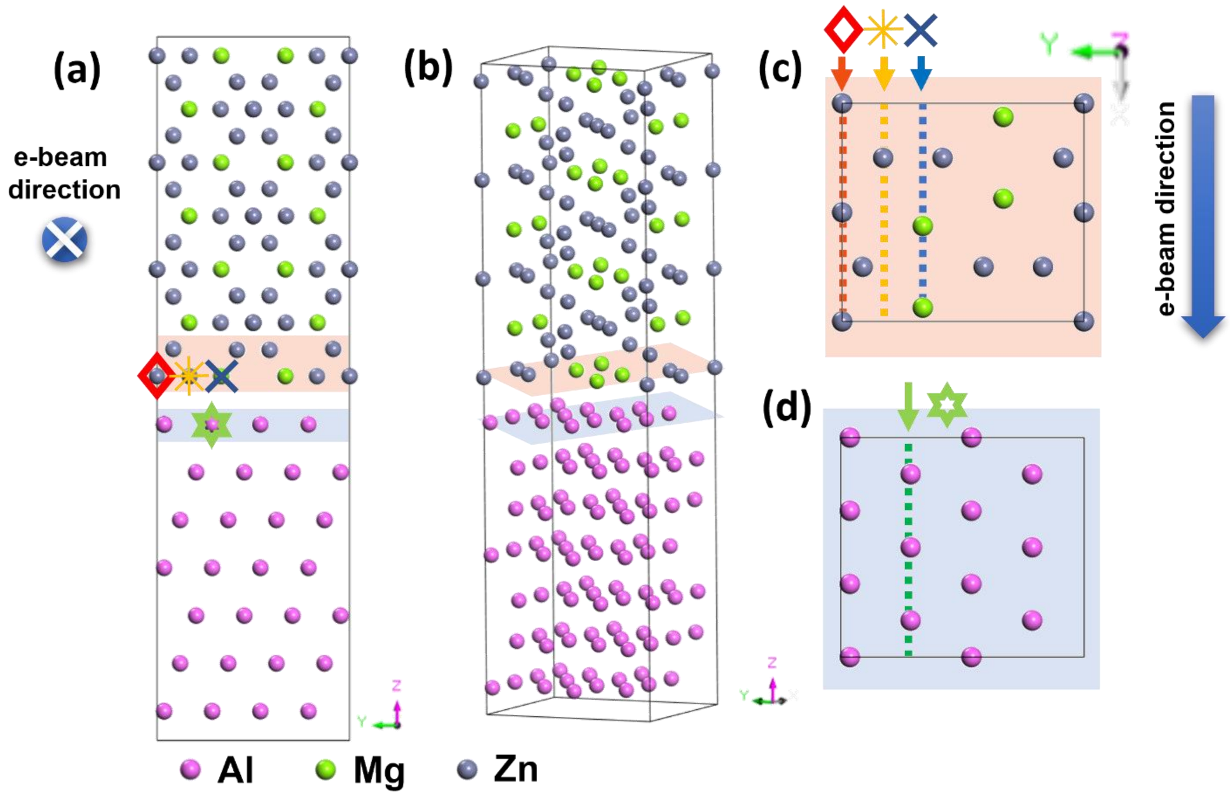

Fig. S2. Schematic diagram of atomic model of η precipitates. (a) and (b) η precipitates shown in different directions. (c) XY plane of orange background area (η_4 precipitate) in (a). (d) XY plane of blue background area (interface) in (a).

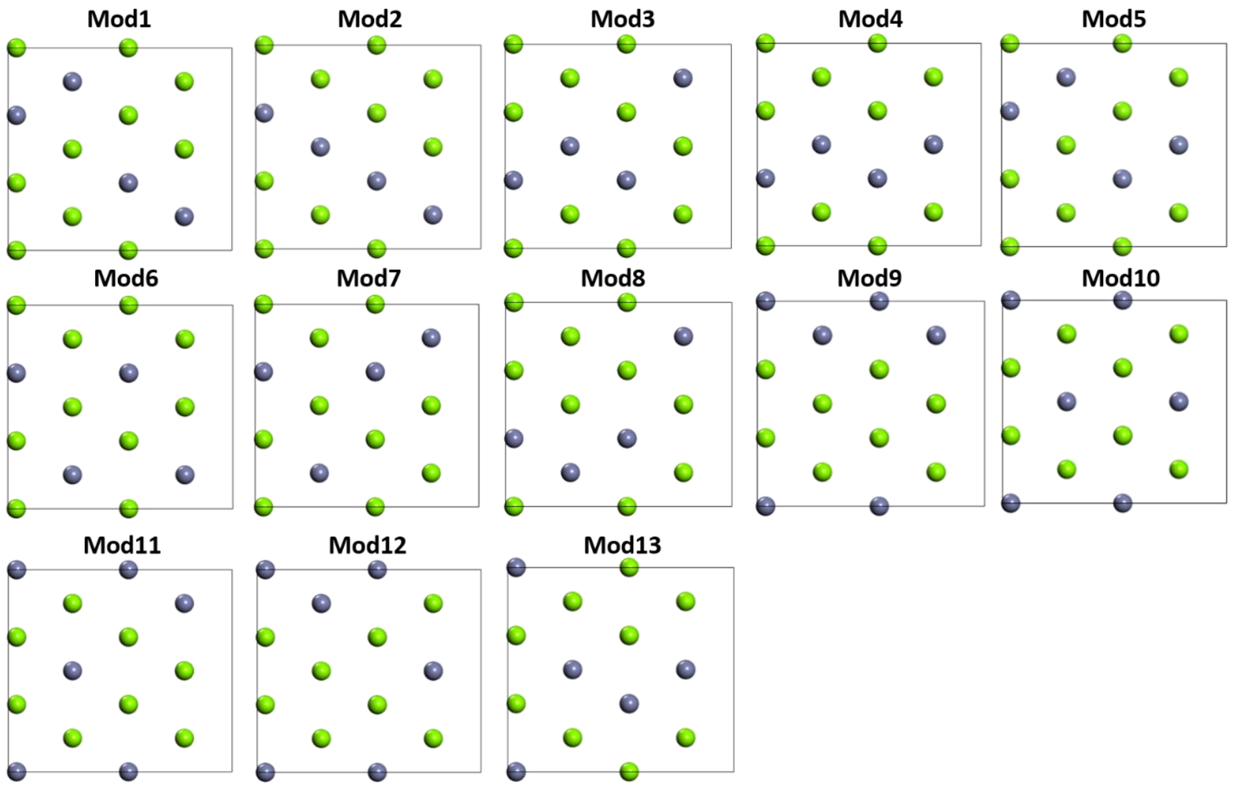

Fig. S3. Possible interface layer models for first principle calculation.

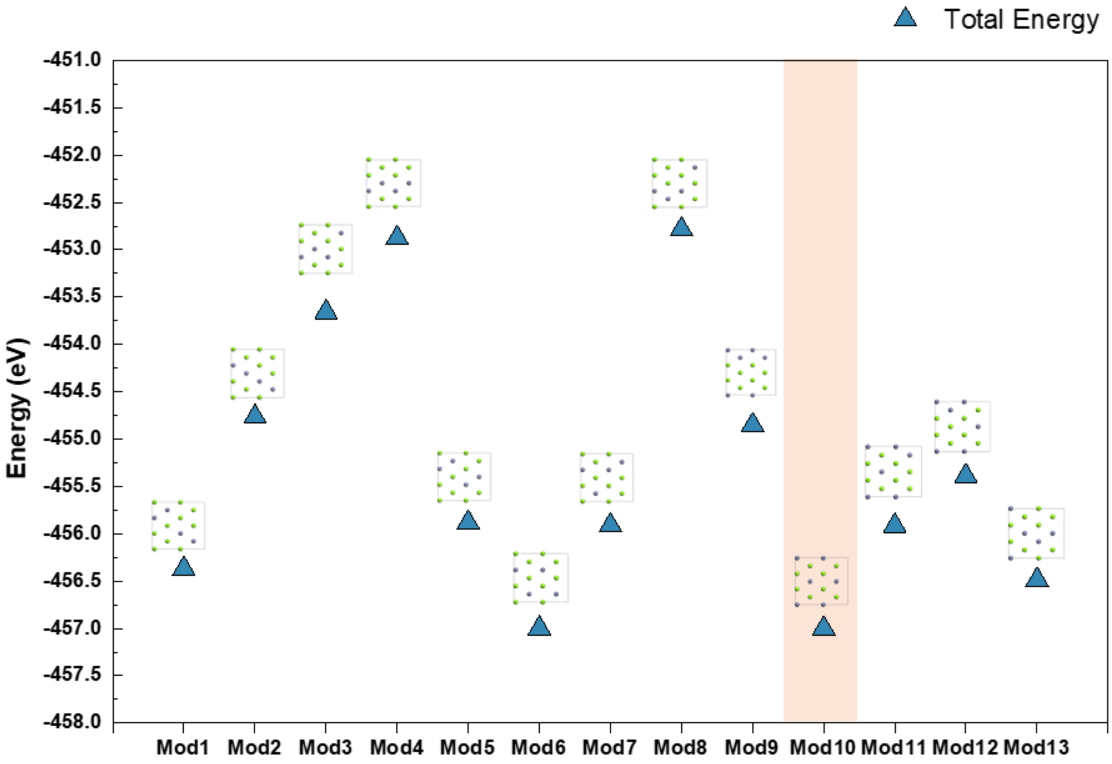

Fig. S4. Calculated total energies of possible structures having η_4/Al interface.

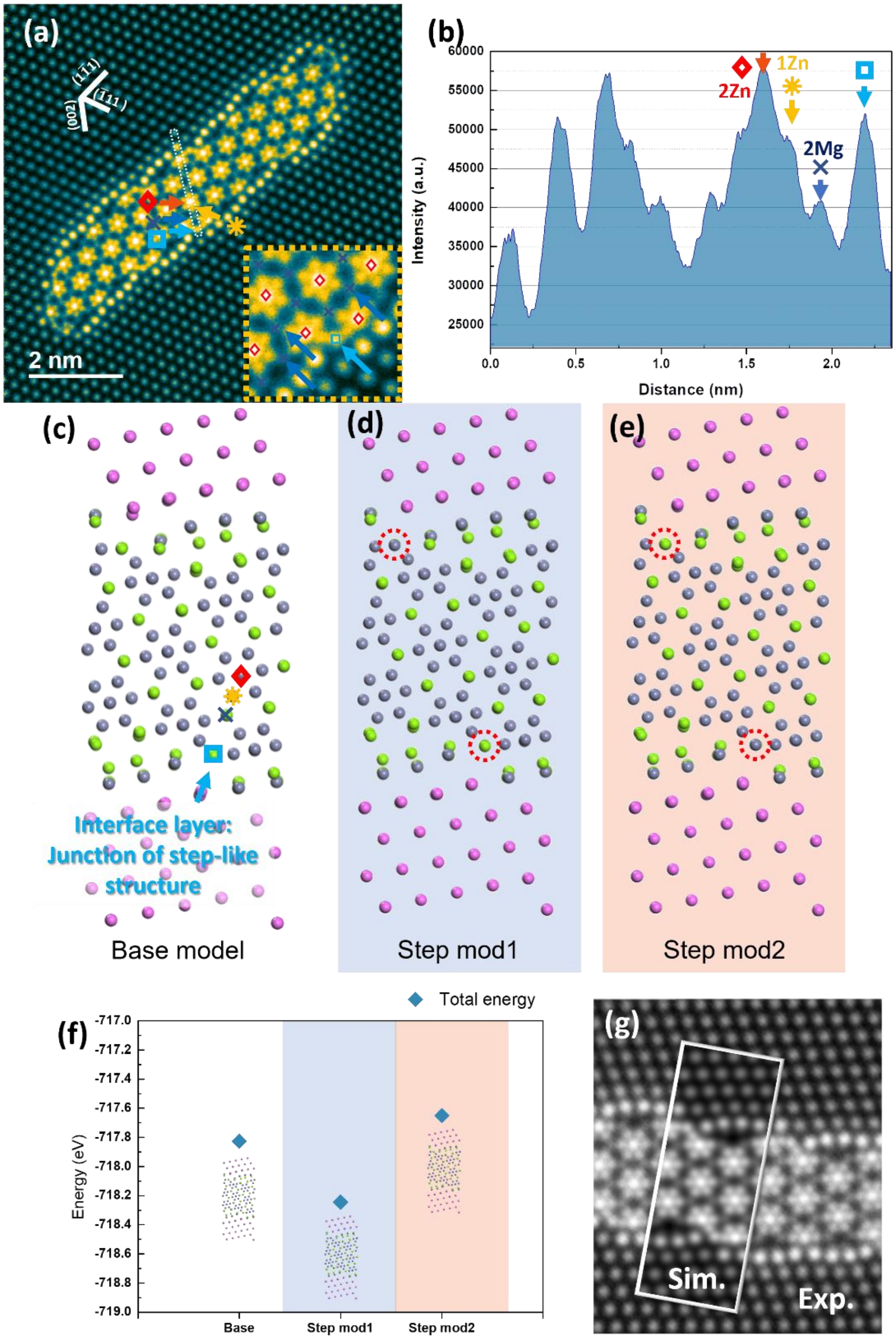

Fig. S5. Possible η_4 considering its step-like structures. (a) HAADF-STEM image of η_4 . Yellow box is enlarged image of the interface especially focused on the stepped shape. Blue arrow indicates 2Mg site, and cyan arrow indicates the stepped interface site. (b) Intensity profile of white dotted box in (a). (c)-(e) Schematic diagram of Base model, Step mod1 and Step mod2. (f) Calculated total energies of 3 step-like structures. (g) Simulated HAADF-STEM image of Step mod1 along $[0001]_\eta$ direction and experimentally observed HAADF-STEM image.

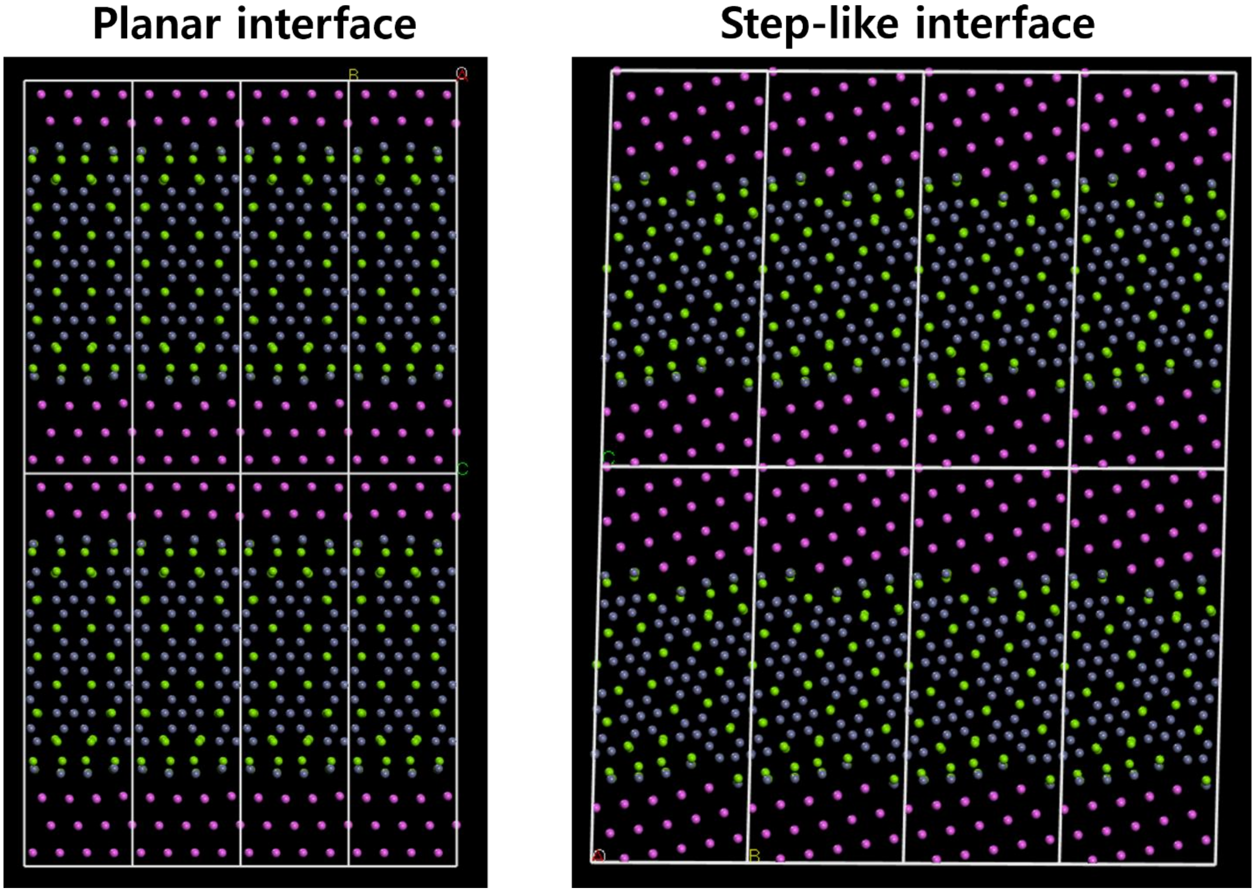

Fig. S6. Periodicity of planar interface and step-like interface model. The superlattice is indicated with white lines.

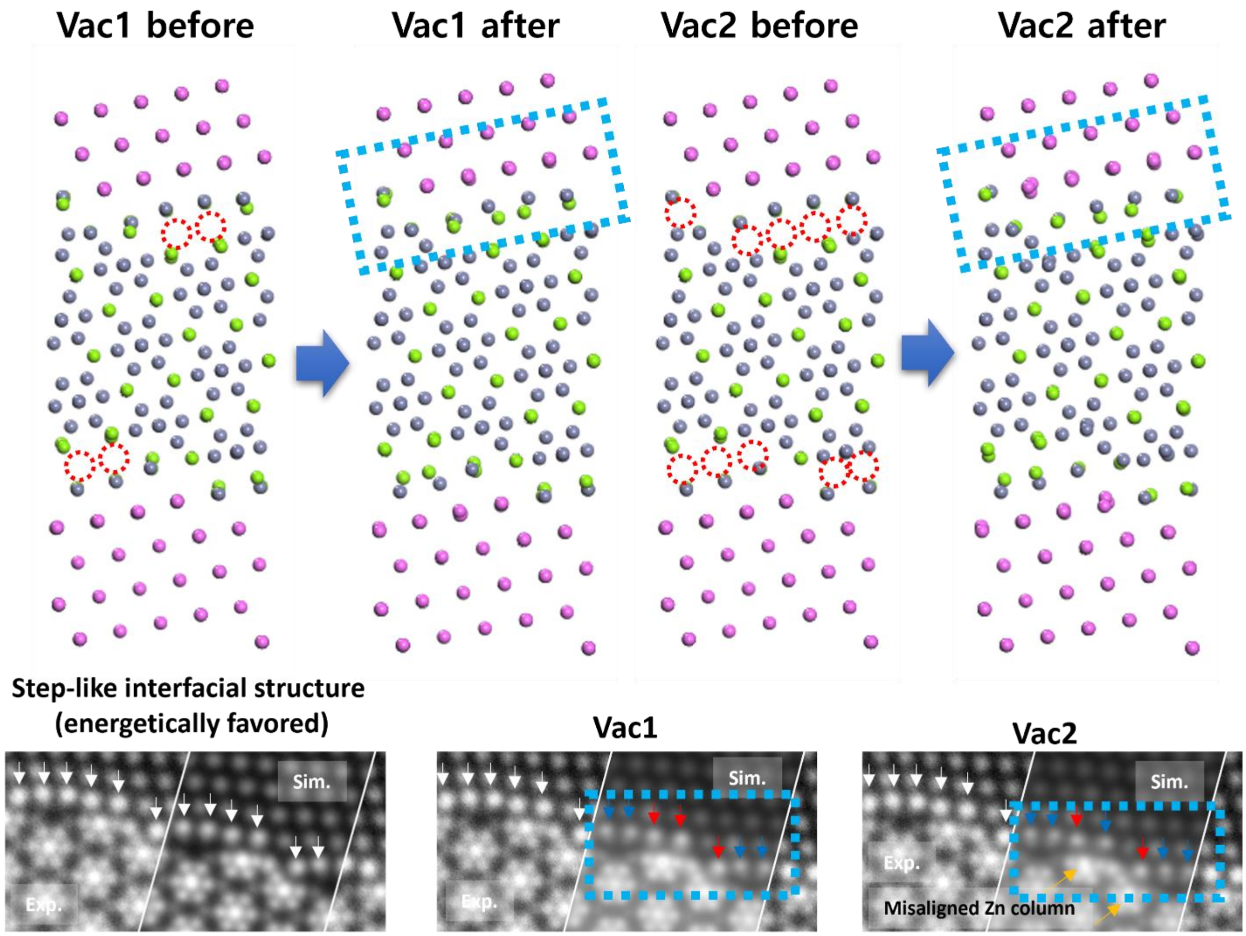

Fig. S7. Schematic model considering the effect of vacancy. Red dotted circles indicate the site of vacancy. The Vac1 model has 4 vacancies and the Vac2 model has 10 vacancies at the interface of η_4/Al. HAADF-STEM image and corresponding simulation results of each model are shown inside the white boxes. Blue dotted boxes represent same area of schematic model and simulation results. The white arrows shows that the intensity of the bridge-shaped interfacial layer is uniform in both experiment and step-like interfacial structure model simulation. The blue arrows indicate relatively low intensity state and the red arrows indicate relatively high intensity state. Both Vac1 and Vac2 model has inhomogeneous interfacial structure intensity distribution.

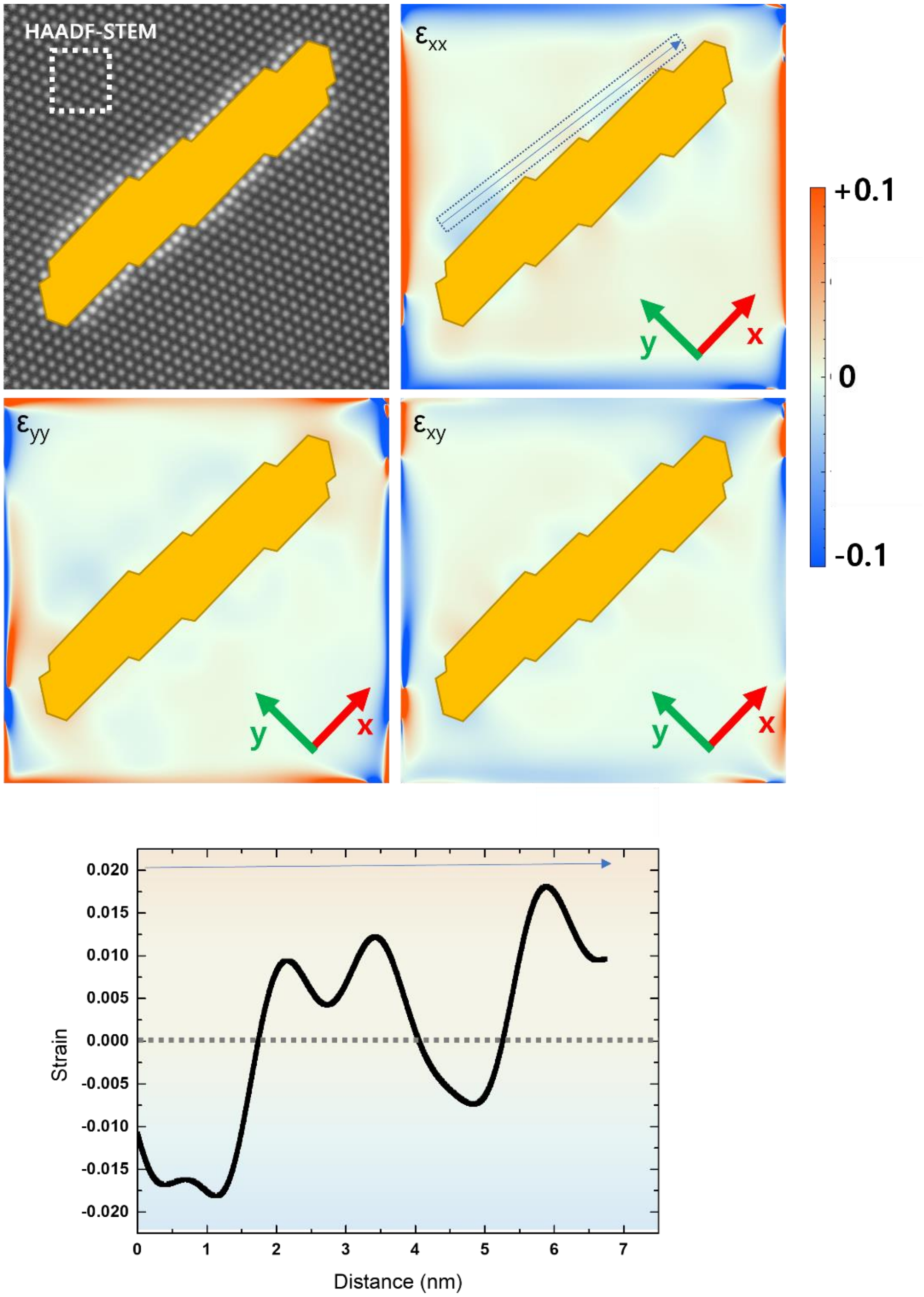

Fig. S8. GPA analysis using HAADF-STEM image of η_4/Al. The white dotted box indicates the reference area.

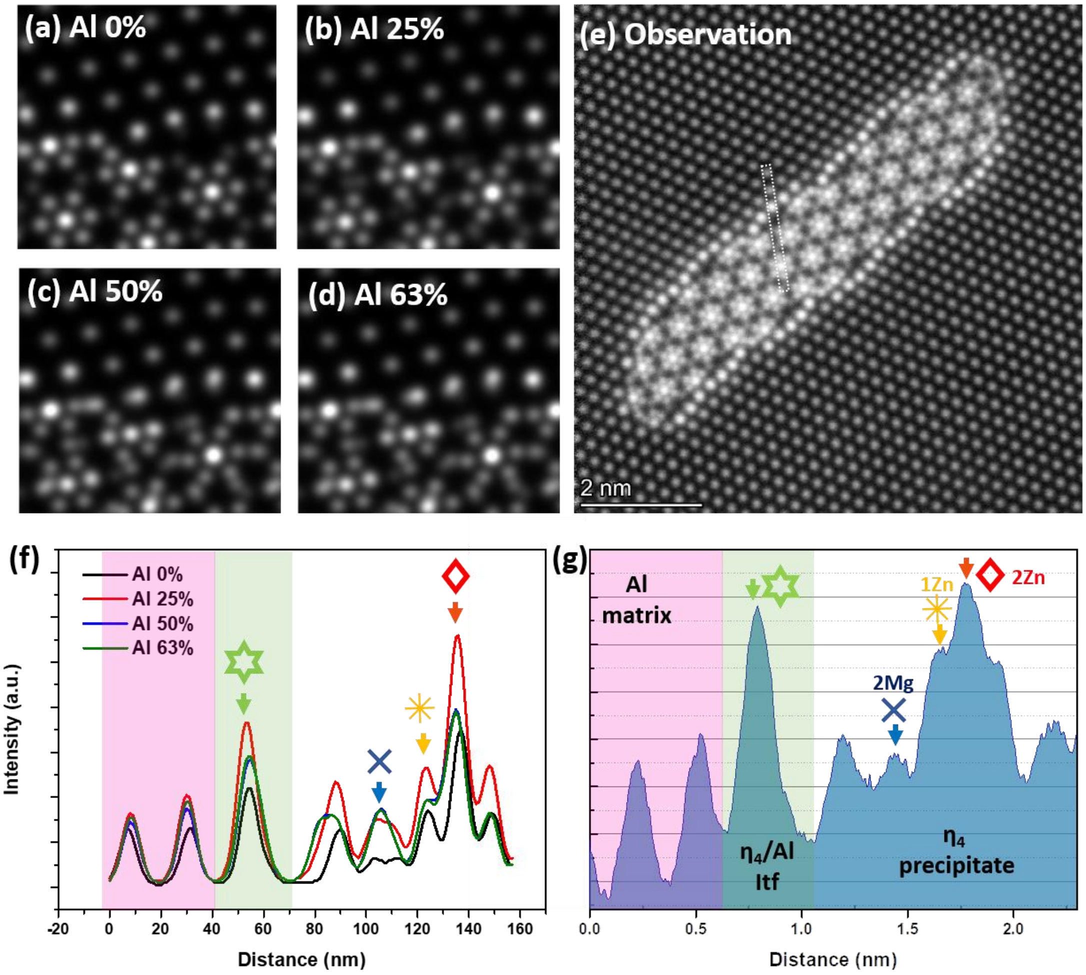

Fig. S9. Effect of Al matrix on quantitative analysis along the e-beam direction.

(a)-(d) STEM simulation result of η_4 with Al matrix. The proportion of Al is (a) 0 % (b) 25 % (c) 50 % (d) 63 % along the e-beam direction. (e) STEM observation result of η_4 with Al. (f) Corresponding intensity profile of (a) to (d) with the same black dotted box area in fig. 3(a). (g) Corresponding intensity profile of (e) from the white dotted box area. (f) and (g) indicates the intensity profile from the same spot of η_4 with Al matrix.

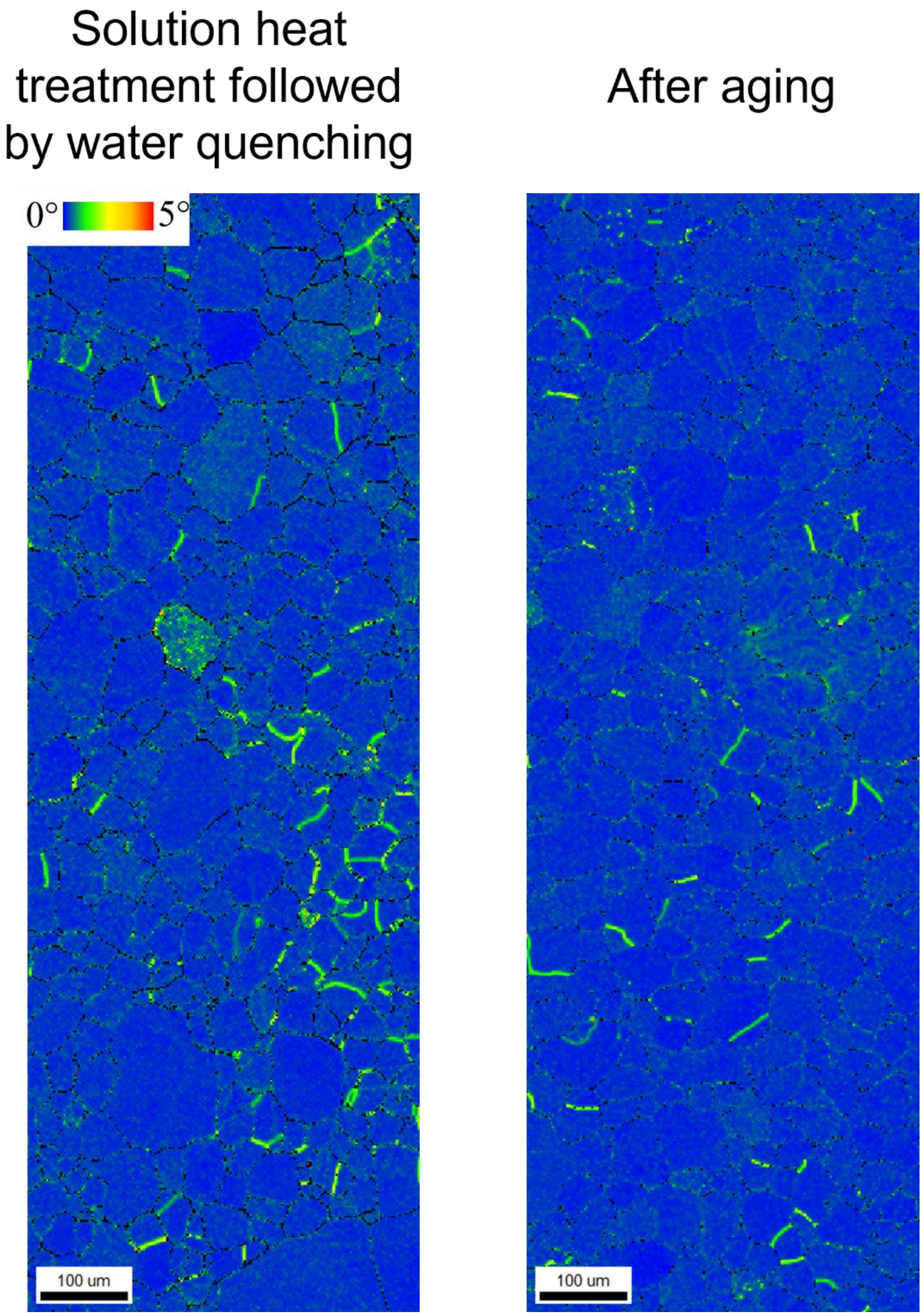

Fig. S10. Kernel average misorientation map of AA7000 alloy after water quenching. The average KAM value of the solution heat treatment followed by water quenching specimen was 0.32 ± 0.22, and the average KAM value of the after aging specimen was 0.28 ± 0.17. All types of KAM values were first-nearest neighbors.
